# Bromide and Chloride Ionic Liquids Applied to Enhance the Vulcanization and Performance of Natural Rubber Biocomposites Filled with Nanosized Silica

**DOI:** 10.3390/nano12071209

**Published:** 2022-04-04

**Authors:** Magdalena Maciejewska, Anna Sowińska-Baranowska

**Affiliations:** Department of Chemistry, Institute of Polymer and Dye Technology, Lodz University of Technology, Stefanowskiego Street 16, 90-537 Lodz, Poland

**Keywords:** ionic liquids, vulcanization, natural rubber (NR), nanosized silica, composites

## Abstract

In this study, the possibility of using ionic liquids (ILs) as auxiliary substances improving the vulcanization and physicochemical properties of natural rubber (NR) biocomposites filled with nanosized silica was investigated. Hence, the influence of ILs with bromide and chloride anions and various cations, i.e., alkylimidazolium, alkylpyrrolidinium and alkylpiperidinium cation, on the curing characteristics and crosslink density of NR compounds was determined. Furthermore, the effect of nanosized silica and ILs on the functional properties of the obtained vulcanizates, including mechanical properties under static and dynamic conditions, hardness, thermal stability and resistance to thermo-oxidative aging, were explored. Applying nanosized silica improved the processing safety of NR compounds but significantly increased the optimal vulcanization time compared to the unfilled rubber. ILs significantly improved the cure characteristics of NR compounds by increasing the rate of vulcanization and the crosslink density of NR biocomposites. Consequently, the tensile strength and hardness of the vulcanizates significantly increased compared to that without ILs. Moreover, the use of nanosized silica and ILs had a favorable impact on the thermal stability of the vulcanizates and their resistance to prolonged thermo-oxidation.

## 1. Introduction

Environmental sustainability based on the use of renewable resources in a manner that satisfies present-day demands is one of the currently accepted technology concepts [1]. Therefore, researchers are trying to look for technological solutions concerning the production of biocomposites that will meet specific requirements for a given application. A renewable biobased polymer that is widely used in a wide range of applications is natural rubber (NR).

Rubber blends prepared from NR are easily subjected to many technological processes, i.e., calendering and extrusion [2,3]. Vulcanized NR has good performance properties, i.e., exceptional elasticity, flexibility, resistance to abrasion and effective heat dissipation [3,4,5,6]. NR vulcanizates, even those without reinforcing fillers, are characterized by a high tensile strength, which is due to the crystallization induced by stretching [7]. Due to the tensile strength being better than that of synthetic rubbers and good thermal resistance, NR is widely used in elastomer technology, e.g., in the production of tires for high performance vehicles such as trucks, racing cars, buses or airplanes [8,9]. It is also used in the electrical industry [10,11], for medical devices and as a raw material to produce surgical gloves [12]. Although the NR elastomer and its composites have been used for years, they are still popular among the researchers.

On the other hand, despite the attractive properties and good processability, fillers, especially the reinforcing ones, are used to obtain NR composites suitable for a variety of the commercial applications. As conventional reinforcing fillers in NR processing, nonrenewable inorganic compounds such as carbon black, calcium carbonate, silica and clays are widely used [13,14,15,16,17]. Due to some advantages, e.g., low cost and biodegradability, these nonrenewable fillers are often replaced by renewable organic fillers derived from plants and animals from natural resources [18,19,20]. Raw renewable organic fillers are often sourced from plant or animal wastes, i.e., straw, wood flour, coir fiber, crab and shrimp shells [20,21,22]. Cellulose extracted from plants or agricultural wastes and chitin or chitosan derived from crab and shrimp shells have been reported to be a good reinforcement for NR composites [23,24,25,26]. However, these renewable organic fillers are not always able to provide the properties of vulcanizates as good as those obtained using common nonrenewable fillers such as silica and carbon black. Rattanasom et al. [27] reported that NR vulcanizates filled with conventional fillers and clay were characterized by satisfied properties. However, it was observed that despite the same hardness, vulcanizates filled with carbon black achieved better overall mechanical properties than the clay-filled or silica-filled vulcanizates, respectively. The authors considered that this was the effect of the poor dispersion of the clay and silica in the elastomer matrix and the low crosslink density of the vulcanizates containing these fillers.

NR elastomer, having a very high molecular weight, becomes highly viscous during melting and processing. Applying a large amount of filler such as silica or carbon black during compounding leads to an additional increase in the viscosity of NR compounds, which makes processing difficult. One approach to decrease the viscosity and to improve the processing of NR composites is the addition of processing aids and some additives that improve the dispersion of solid ingredients in the elastomer matrix [28,29]. Boonmahitthisud et al. [30] summarized several significant solutions to achieve “green” NR biocomposites, i.e., applying renewable organic fillers, the use of effective zinc oxide activators for sulfur vulcanization, the development of a harmless preservative system for NR latex and the application of different biobased processing aids alternatively to the commercial processing aids such as petroleum-based oils including naphthenic, paraffinic or aromatic oils, which are the most extensively used due to their low price and good compatibility with most of rubbers. However, because aromatic oil contains high contents of polycyclic aromatics, it is classified as a carcinogenic agent. As a consequence, new alternative and environmentally friendly additives are still being searched for, which facilitates processing and improves the properties of NR composites, especially those containing a significant amount of the filler. Ionic liquids (ILs) seem to be good potential candidates for alternative processing aids for NR composites.

Ionic liquids (ILs) have been a broad prospect in the field of green catalysis for years due to their low toxicity, negligible vapor pressure, excellent chemical and thermal stability and the possibility of designing them for special applications [31,32,33]. Due to the high affinity of ILs toward different fillers [34,35,36], they can interact with them via hydrogen bonding, van der Waals forces, cation −π− or delocalized electron interactions [37,38]. Moreover, ILs have been reported to improve the solubility of active sulfurating agents formed during the vulcanization of rubber compounds and hence to increase the crosslinking degree of elastomer. In addition, ILs affected the dispersion of fillers and zinc oxide in the elastomer matrix, improving the properties of elastomer composites [39]. Hussain et al. [40] emphasized that the dispersion of nanoparticles in the elastomer matrix is essential for the good performance of rubber composites. Thus, ILs with silanes were used to produce silica-filled butadiene rubber vulcanizates. Performed studies showed that the replacement of a half of silane by ILs improved the dispersion of zinc oxide and silica in the elastomer matrix. Moreover, ILs were reported to enhance the crosslinking efficiency probably by reducing the adsorption of sulfur and other components of the crosslinking system on the surface of silica. Furthermore, Sowinska et al. [41] proved that ILs with bis(trifluoromethylsulfonyl) imide (TFSI) anion could be employed to fine-tune the cure characteristics and performance of NR composites containing silica. The activity of TFSI ILs in the vulcanization and the crosslink density of NR composites strongly depended on the structure of the ILs cation (i.e., pyrrolidinium, ammonium, sulfonium). No less important was the length of alkyl chain present in the cation of ILs. On the other hand, TFSI is an organic anion with a weak coordinating ability that has a significant impact on the properties of ILs such as solvent abilities, interaction abilities (e.g., hydrogen bonding), miscibility and melting point [42]. These properties of ILs can affect their activity in the elastomer composites. Therefore, considering NR composites, it is reasonable to explore the applications of ILs with an anion of a different nature from that of TFSI and thus containing simple, inorganic anions.

Therefore, in this study, we applied ILs with simple halogen anions and different cations to improve the cure characteristics and physico-chemical properties of NR composites filled with nanosized silica. ILs with bromide and chloride anions and alkylimidazolium, alkylpyrrolidinium and alkylpiperidinium cations with different lengths of alkyl substituents were employed to boost the sulfur vulcanization of NR composites and to enhance their performance, such as their mechanical properties, resistance to prolonged thermo-oxidation and thermal stability. Efforts have been made to find the relationship between the structure of ILs and their influence on vulcanization parameters and composites properties. To our knowledge, no systematic research has been carried out so far on the activity of ILs with bromide and chloride anions and cations of different structures in NR composites filled with the nanosized silica.

## 2. Materials and Methods

### 2.1. Materials

**Figure 1:**
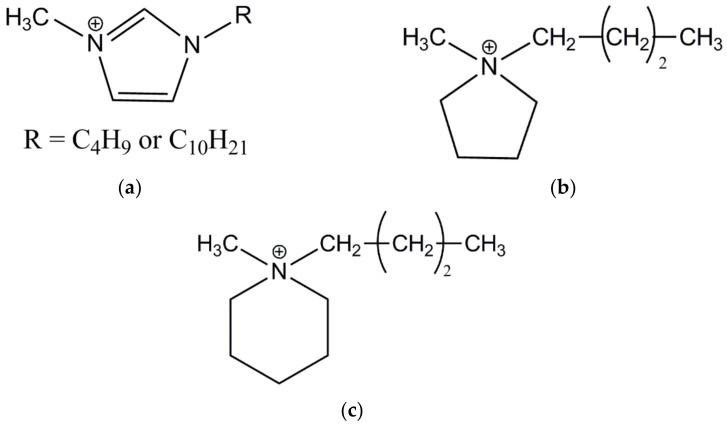
Structure of IL cations (R-alkyl substituent): (**a**) imidazolium; (**b**) pyrrolidinium; (**c**) piperidinium.

Natural rubber (NR, RSS1 type, *cis*-1,4-polyisoprene) with a density of 0.93–0.988 g/cm^3^ was obtained from Torimex Chemicals, Lodz, Poland. A conventional crosslinking system containing sulfur as a crosslinker (Siarkopol, Tarnobrzeg, Poland) in the presence of a vulcanization accelerator, i.e., 2-mercaptobenzothiazole (MBT), purchased from Sigma-Aldrich, Schelldorf, Germany, was applied. Zinc oxide (ZnO) with a specific surface area of 10 m^2^·g^−1^ (Huta Bedzin, Bedzin, Poland) together with stearic acid (St.A, Sigma-Aldrich, Schelldorf, Germany) were used to activate the vulcanization. Silica AEROSIL^®^ 380 (A380) supplied by Evonik Industries (Essen, Germany) was used as a filler. Additionally, eight ionic liquids (ILs) with characteristics given in Table 1 were applied to enhance the vulcanization and properties of NR composites. ILs were provided by IoLiTec Ionic Liquids Technologies GmbH, Heilbronn, Germany. These ILs consist of bromide or chloride anion and different cations with butyl or decyl substituents. The general structures of ILs cations are given in Figure 1.

### 2.2. Preparation and Characterization of NR Compounds Filled with Nanosized Silica

NR compounds were prepared according to the general formulations presented in Table 2 as parts per hundred of rubber (phr). The mass of a particular component of the rubber compound was given in grams per 100 g of pure rubber. A laboratory two-roll mill (rolls dimensions: D = 200 mm, L = 450 mm) produced by Bridge, Rochdale, UK was adopted for compounding. The friction and the width of the gap between rollers were set for 1–1.2 and 1.5–3 mm, respectively, whereas the rotational speed of the front roll was 16 min^−1^. During NR composites preparation, the average temperature of the rolls was approximately 30 °C.

Using the optimal vulcanization times determined during rheometric measurements, NR composites were vulcanized at 160 °C and at 15 MPa pressure. The cure characteristics of NR compounds were investigated at 160 °C according to the ISO 6502 [43] standard procedures. The rotorless D-RPA 3000 rheometer (MonTech, Buchen, Germany) was employed to perform rheometric measurements. The optimal vulcanization time (t_90_) was determined as the time for rheometric torque to reach 90% of the maximum achievable torque value as given by Equation (1), where ∆*S* is the torque increase during the rheometric test, calculated as the difference between the maximum (*S_max_*) and the minimum (*S_min_*) torque. Adapting a similar equation, the scorch time (t_02_) was determined.

(1) $$S_{90}=0.90\Delta S+S_{min},$$

To examine the effect of the structure of ILs on the temperatures and the enthalpy of NR vulcanization, a differential scanning calorimeter DSC1 (Mettler Toledo, Greifensee, Switzerland) equipped with a STARe software (Version 10, 2010, Greifensee, Switzerland) was investigated. The onset temperature of the peak corresponding to vulcanization was determined according to the ISO 11357-1 [44] standard. Liquid nitrogen was used as a cooling agent, whereas the nitrogen (flow rate 80 mL/min) was applied as the protective gas. The analysis was performed for samples of rubber compounds with a mass of approximately 10 mg. Before measurement, the samples were placed in a hermetically sealed aluminum crucible with a capacity of 40 µL. During DSC measurement, the sample was heated from −100 °C to 250 °C, with a heating rate of 10 K/min.

The equilibrium swelling method was adopted to determine the crosslink density of NR vulcanizates. Toluene was used as a solvent. The crosslink density was determined according to the procedure given in the ISO 1817 [45] standard and using the Flory–Rehner equation [46]. Small pieces of the vulcanizates with masses in the range of 20–30 mg were swollen in toluene for 48 h. Subsequently, after removing the solvent and weighing the samples, they were dried at 50 °C for another 48 h. At the end, the dried samples were reweighed. The crosslink density was calculated using the Huggins parameter of NR-toluene interaction (*χ*) given by Equation (2), where *V_r_* is the volume of the elastomer fraction in swollen gel [47].

(2) $$\chi =0.780+0.404V_{r},$$

The Zwick Roell 1435 (Ulm, Germany) universal testing machine was employed to investigate the tensile properties of NR vulcanizates. Tensile tests were carried out in static conditions according to the ISO 37 [48] standard procedure. Five dumb-bell-shaped samples were used from each tested vulcanizate. Their thickness was approximately 1 mm and the width of their measuring section was 4 mm. The crosshead speed during measurements was 500 mm/min.

The hardness of NR vulcanizates was measured by a Zwick Roell 3105 (Ulm, Germany) hardness tester. Measurements were carried out using Shore’s method and following the standard ISO 868 [49]. Disc-shaped specimens were used for hardness measurements.

The thermo-oxidative aging of NR vulcanizates was carried out according to the procedure described in ISO 188 standard [50]. Plates of the vulcanized rubber compounds with a thickness of approximately 1 mm were placed in a drying chamber (Binder, Tuttlingen, Germany) at a temperature of 70 °C. The theromo-oxidative aging was continued for ten days (240 h). To establish the resistance of the vulcanizates to prolonged thermo-oxidation, their crosslink densities, tensile properties and hardness were examined and compared with the values obtained for non-aged vulcanizates. The aging coefficient (*A_f_*), which quantifies the resistance of material to prolonged thermo-oxidation, was determined using Equation (3) [51], where *TS* is the tensile strength of vulcanizates and *E_b_* is the elongation at break.

(3) $$A_{f}=\frac{{\left(E_{b}\times TS\right)}_{after\;aging}}{{\left(E_{b}\times TS\right)}_{before\;aging}},$$

Additionally, to more accurately illustrate the differences between crosslink density before and after the aging process, the value of the crosslink density increase (%) given by Equation (4) was calculated.

(4) $$\Delta \nu_{t}=\frac{\nu_{t_{after\;aging}}-\nu_{t_{before\;aging}}}{\nu_{t_{before\;aging}}}\times 100,$$

Thermogravimetric (TG) measurements were performed to study the influence of ILs on the thermal stability of NR vulcanizates. A thermogravimetry/differential scanning calorimetry TGA/DSC1 analyzer (Mettler Toledo, Greifensee, Switzerland) was used to conduct measurements for NR vulcanizates. Before measurement, a small piece of each vulcanizate with a mass of approximately 10 mg was placed in the open alumina crucibles with a capacity of 70 µL. Measurements were carried out using two measuring segments. In the first segment of measurement, the specimen was heated in a temperature range of 25–600 °C in argon atmosphere (50 mL/min) with a heating rate of 20 K/min. In the second measuring segment, the gas was changed into air (50 mL/min), and heating was continued up to 800 °C with the same heating rate.

A DMA/SDTA861e analyzer (Mettler Toledo, Greifensee, Switzerland) was employed to perform dynamic mechanical analysis (DMA). Measurements were carried out in tension mode. During DMA tests, samples of the vulcanizates were heated in a temperature range of −100–80 °C with a heating rate of 3 K/min. The frequency of strain oscillation during the measurements was 1 Hz, whereas the strain amplitude was 4 µm. The strip-shaped specimens with a width of 4 mm, length of 10.5 mm and thickness of approximately 1 mm were used for DMA measurements. The glass transition temperature (T_g_) of the elastomer was determined from the maximum of the tan δ = f(T) curve, where tan δ is the mechanical loss factor and T is the measurement temperature. Liquid nitrogen was applied as a cooling medium.

## 3. Results and Discussion

### 3.1. Effect of Ionic Liquids on Cure Characteristics and Crosslink Density of NR Composites Filled with Nanosized Silica

In the first step of the studies, the rheometric properties of NR compounds were investigated to determine the influence of the ILs structure on the curing parameters. The rheometric tests were carried out at 160 °C, and the results are summarized in Table 3.

The minimum rheometric torque (S_min_) is a measure of the viscosity of the uncrosslinked rubber compound. The value of S_min_ for the unfilled NR compound was 0.3 dNm. As expected, the addition of the nanosized silica as a filler significantly increased the viscosity of the uncured NR compound, as evidenced by the increase in S_min_ to 6.0 dNm. Considering the measurement error, ILs did not significantly affect the S_min_ as compared to the rubber compound without ionic liquid.

The maximum torque (S_max_) depends on the crosslinking degree of the elastomer and also on the hydrodynamic effect of the filler and its activity. The unfilled NR compound showed a S_max_ value of 5.0 dNm. The addition of nanosized silica caused a significant increase in the S_max_ in relation to the unfilled benchmark. The S_max_ of the rubber compound filled with nanosized silica was 11.4 dNm. The considerable increment of S_max_ was due to the hydrodynamic effect of the nanosized silica, as well as the filler-elastomer and filler-filler interactions. Introduction of the rigid phase of the filler network into the soft elastomer matrix considerably hindered the mobility of elastomer chains. On the other hand, ILs increased the S_max_ as compared to the unfilled reference compound, which could result from the improved filler-polymer interactions as well as from the enhanced degree of elastomer crosslinking. The highest S_max_ was observed for NR compounds containing imidazolium ILs, especially those with decyl chains, whereas the lowest values of S_max_ were determined for NR compounds with piperidinium ILs.

The rheometric torque increase (∆S) can be considered as a measure of the crosslinking degree of elastomer. The addition of nanosized silica did not significantly affect the torque increase during vulcanization as compared to the unfilled benchmark (ΔS of approximately 5 dNm). On the other hand, ILs improved the torque increase of the silica-filled NR compounds; thus, they affected the crosslinking degree of the elastomer. NR compounds with imidazolium ILs containing decyl chains were characterized by the highest ΔS values (ΔS of 9.2 dNm and 11.8 dNm for DmiBr and DmiCl, respectively), whereas those with piperidinium ILs showed the lowest ΔS of approximately 7 dNm.

The scorch time (t_02_) indicates the safety of the processing of rubber composites at a certain temperature. The longer the t_02_, the more secure the processing; thus, the rubber compound can be processed and formed without the risk of scorching. The unfilled rubber compound exhibited a t_02_ of 1 min, whereas the scorch time for the NR compound filled with silica was extended to 2 min, which resulted in the improved safety of the processing. However, ILs regardless of their structure reduced the t_02_ of the silica-filled NR compounds to 1 min, along with the value characteristic for the unfilled NR.

The optimal vulcanization time (t_90_) is a very important parameter from a technological and economic point of view. Shorter vulcanization times are more industrially preferred as they allow for the reduction of the total cost of obtaining final rubber products by reducing the cost of rubber compounds vulcanization. The t_90_ for the unfilled benchmark was 2 min, whereas the NR compound filled with the nanosized silica was characterized by six times longer t_90_ as compared to the unfilled benchmark. This was probably due to the ability of the silica surface to adsorb the crosslinking system, especially vulcanization accelerators, which was confirmed by Kosmalska et al. [52], Rattanasom et al. [53] and Zaborski et al. [54]. Moreover, silica A380 has an acidic pH in the range of 3.7–4.5, whereas vulcanization prefers alkaline conditions to proceed faster and more efficiently [55]. Therefore, using nanosized silica A380 prolonged the t_90_ of the NR compound compared to the unfilled benchmark. Most importantly, ILs reduced the t_90_ to 1–3 min, along with the value comparable to that of the unfilled benchmark. A similar effect of ILs on the t_90_ was observed in our previous work [56]. According to Hussain et al. [40] the positive influence of ILs on the curing rate and efficiency could be due to the preferential adsorption of ILs on the silica surface, which reduces its ability to adsorb the accelerator. In our previous studies [57] we have also confirmed that ILs can be successfully immobilized on the surface of fillers, including the silica of different particle sizes and specific surface areas. Moreover, ILs were reported to support the vulcanization of various rubbers, probably due to their catalytic activity in the interface reactions, including crosslinking reactions [58,59,60,61]. The surface activity of ILs has been commonly used in some synthetic, catalytic and separation processes, and ILs are considered as phase-transfer catalysts [62,63].

**Figure 2:**
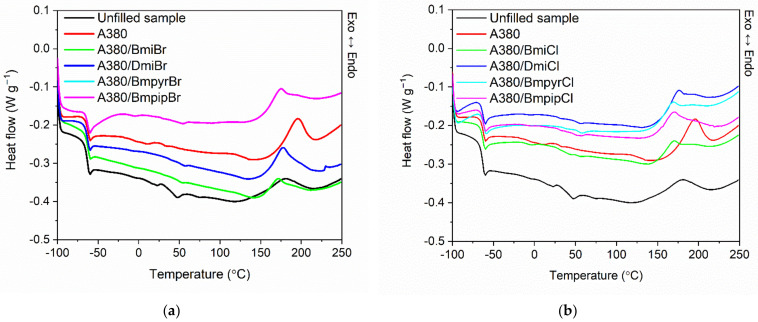
Differential scanning calorimetry (DSC) curves of the silica-filled NR compounds containing ILs with: (**a**) bromide anion; (**b**) chloride anion.

Having established the influence of ILs on the cure characteristics of the silica-filled NR compounds, we then studied their impact on the crosslinking temperature and the enthalpy of crosslinking reactions using differential scanning calorimetry (DSC). The effect of ILs on the glass transition temperature (T_g_) of the NR elastomer was explored as well. The DSC curves of NR compounds are given in Figure 2, and the results are summarized in Table 4.

By analyzing the differential scanning calorimetry (DSC) curves presented in Figure 2, a step on the DSC curves was observed, which resulted from the change in the heat capacity (∆C_p_) due to the glass transition of NR. A midpoint of this step corresponds to T_g_, which is an important parameter for establishing the service temperature range of elastomeric products. The T_g_ of NR determined for the unfilled benchmark was approximately −64 °C. The addition of nanosized silica, as well as the structure of ILs, did not significantly affect the T_g_ of the elastomer, which was approximately from −64 to −63 °C, which is typical for NR elastomers [41]. The incorporation of the nanosized silica considerably reduced the ∆C_p_. It resulted from the increased stiffness of the material due to the restricted mobility of the elastomer chains by the filler network. ILs had no significant impact on the ∆C_p_ as compared to the NR composite without ionic liquid.

By analyzing the DSC plots, the crosslinking of NR compounds was observed as an exothermic process in the temperature range dependent on the composition of the rubber compounds, i.e., the addition of the filler and ILs. Crosslinking of the unfilled NR compound proceeded in the temperature range of 146–208 °C, with an enthalpy of crosslinking (∆H_cross_) of approximately 9.0 J/g. The addition of silica nanoparticles increased the temperature range and the enthalpy of crosslinking. The silica-filled NR was characterized by a 25 °C higher onset crosslinking temperature compared to the unfilled NR, which could result from the accelerator absorption on the silica surface [52,53]. Moreover, the crosslinking of the silica-filled rubber compound proceeded in a wider range of temperatures compared to the unfilled NR. The endset crosslinking temperature was approximately 2 °C higher than that of the unfilled benchmark, which was probably due to the fact that the filler network hampers the diffusion of heat and the components of the curing system through the elastomer matrix during vulcanization [53,55,64]. Considering the measurement error, nanosized silica did not significantly affect the enthalpy of crosslinking process compared to the unfilled NR.

Most importantly, ILs significantly lowered both the onset and the endset temperature of crosslinking compared to the silica-filled rubber compound without ILs. This was probably due to the postulated adsorption of ILs on the silica surface, which decreased the ability of the filler surface to adsorb the curing system, especially the accelerator. The structure of ILs, i.e., the type of anion and cation, and, more specifically, the length of the alkyl chain in the cation, did not have a significant impact on the range of vulcanization temperature. Regarding the enthalpy of crosslinking, and therefore the amount of heat released during the crosslinking process, most of the ILs reduced ∆H_cross_ to a value comparable to that of the unfilled rubber compound. The only exceptions were imidazolium ILs with a decyl substituent in the cation as well as pyrrolidinium and piperidinium bromides, which slightly increased the amount of heat released during vulcanization compared to the unfilled NR and the rubber compound filled with silica.

Both the rheometric tests and DSC analysis confirmed the beneficial influence of ILs on the cure characteristics of NR compounds filled with the nanosized silica. Therefore, in the next step of the research, the effect of ILs on the crosslink density of NR vulcanizates was explored. The results are shown in Table 5.

It should be noticed that the greatest equilibrium swelling (Q_t_) in toluene was shown by the silica-filled vulcanizate without ILs. Consequently, this vulcanizate exhibited the lowest crosslink density (*ν_t_*). It should be noticed that the adsorption of the curing system on the silica surface reduced its activity and thus the crosslink density of the silica-filled vulcanizate as compared to the unfilled benchmark. On the other hand, vulcanizates with ILs showed lower Q_t_ in comparison with the vulcanizate without ILs. This was due to their significantly higher crosslink density compared to the vulcanizate without ionic liquid. Thus, it was proved that ILs significantly increased the crosslink density of the silica-filled vulcanizates. As mentioned, this was probably due to both the ILs adsorption on the silica surface, which decreased its ability to adsorb the curing system [40,53,54], and the catalytic activity of ILs in the interface crosslinking reactions [58,59,60,61,62]. Vulcanizates containing imidazolium ILs, especially with butyl chain, were characterized by the highest crosslink density as compared to vulcanizates with pyrrolidinium and piperidinium ILs. Taking into account the measurement error, the anion of ILs had no significant influence on the crosslink density of NR vulcanizates.

Having established the impact of nanosized silica and ILs on the vulcanization parameters of NR compounds and the crosslink density of the vulcanizates, we then investigated their effect on the mechanical properties of NR vulcanizates.

### 3.2. Effect of Ionic Liquids on the Tensile Properties and Hardness of NR Composites Filled with Nanosized Silica

Crosslink density is one of the main parameters affecting the performance of vulcanizates, including their tensile properties [65]. As discussed earlier, the addition and the type of ILs affected the crosslink density of the examined vulcanizates and hence could significantly alter their mechanical properties and hardness. In order to study the performance of NR vulcanizates during stretching, we determined their stress at 100% and 300% relative elongation (Se), tensile strength (TS) and elongation at break (E_b_). The results are summarized in Table 6.

Analyzing the data collected in Table 6, the addition of the silica nanoparticles increased the stress at a relative elongation of 100% and 300% (Se_100_ and Se_300_, respectively) compared to the unfilled benchmark. This was due to the restricted mobility of elastomer chains resulting from the filler network created in the elastomer matrix by the silica nanoparticles. ILs did not significantly affect the Se_100_ as compared to vulcanizate without ILs, because Se_100_ depends mainly on the addition and on the type of the filler used, whereas examined vulcanizates contained the same amount of the same silica filler. Vulcanizates containing ILs, especially those with chloride anion, exhibited significantly higher Se_300_ compared to the unfilled benchmark or the silica-filled vulcanizate without ILs. It is commonly known that Se_300_ depends strongly on the crosslink density of the vulcanizates and increases when the crosslink density increases [66]. Therefore, significantly higher Se_300_ of the ILs-containing vulcanizates resulted from their considerably higher crosslink density compared to that of the composite without ionic liquid.

As expected, the addition of nanosized silica and ILs affected the tensile strength (TS) of NR vulcanizates. The unfilled vulcanizate reached a TS of 9.5 MPa, whereas vulcanizate filled with nanosized silica exhibited approximately 4 MPa lower TS compared to the unfilled benchmark. There are several reasons for the deterioration of the TS of NR vulcanizate due to the incorporation of the silica. The first reason is the incompatibility of NR, which is a hydrophobic polymer, with the silica, which, due to the high content of -OH groups, is a highly hydrophilic filler. Therefore, silica nanoparticles weakly interact with NR elastomer and tend to agglomerate, which deteriorates the TS. In addition, the filler network may limit the tendency of NR to the stretch induced crystallization, which is a reason for the good mechanical properties of this elastomer [67]. Moreover, application of nanosized silica significantly decreased the crosslink density of NR vulcanizate as compared to the unfilled benchmark. Consequently, TS of the NR vulcanizate was reduced to approximately 5 MPa. Owing to the beneficial influence of ILs on the vulcanizates crosslink density, their application significantly improved the TS compared to the vulcanizate without ILs. Considering the measurement error, most of the vulcanizates containing ILs showed TS comparable to that of the unfilled benchmark. Moreover, the application of DmiCl and BmpipCl resulted in the improvement of TS by 1.4–1.8 MPa compared to the unfilled NR vulcanizate.

Regarding the elongation at break (E_b_) of vulcanizates, which is a parameter strongly dependent on the filler’s addition and the crosslink density, introducing the nanosized silica or ILs to NR composites reduced the E_b_ by approximately 200% as compared to the unfilled benchmark. This resulted from the significantly reduced mobility of the elastomer chains by the network formed by nanoparticles of silica in the elastomer matrix. Most vulcanizates with ILs were characterized by slightly lower E_b_ than the silica-filled vulcanizate, which correlated with their higher crosslink density compared to the vulcanizate without ILs. The E_b_ of the silica-filled vulcanizates ranged from 565 to 634%, which allows these composites to be used in a variety of potential applications.

The hardness of NR vulcanizates is directly proportional to their crosslink density and also strongly depends on the addition and the type of the filler. As expected, the unfilled vulcanizate was characterized by the lowest hardness of approximately 30 Shore A. The addition of nanosized silica increased the hardness of NR vulcanizates by 22 Shore A compared to the unfilled benchmark. This resulted mainly from the reduction of the mobility of elastomer chains by the filler’s network. A similar effect of the silica on the hardness of vulcanizates was reported by Ulfah et al. [68]. Due to the higher crosslink density, ILs-containing vulcanizates were characterized by 4–9 Shore A higher hardness as compared to the silica-filled vulcanizate without ILs. The hardness of the silica-filled vulcanizates containing ILs ranged from 55 Shore A to 60 Shore A.

### 3.3. Effect of Ionic Liquids on the Dynamic Mechanical Properties of NR Composites Filled with Nanosized Silica

**Figure 3:**
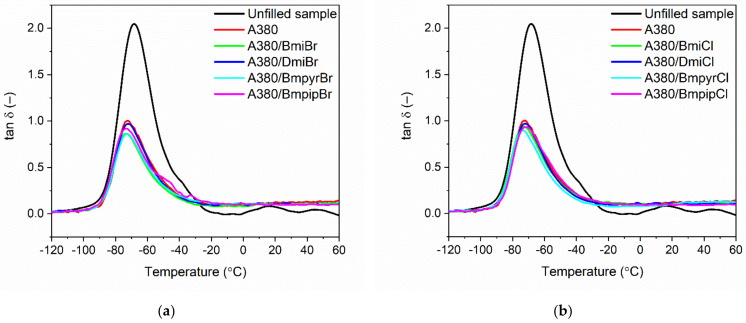
Mechanical loss factor (tan *δ*) curves versus temperature of the silica-filled NR vulcanizates containing ILs with: (**a**) bromide anion; (**b**) chloride anion.

The influence of the ILs on the glass transition of the NR elastomer and its dynamic mechanical behavior was examined using dynamic mechanical analysis (DMA). The mechanical loss factor tan δ was measured as a function of temperature to study the viscoelastic properties of NR vulcanizates. The results are shown in Figure 3 and summarized in Table 7.

Analyzing the DMA curves of NR vulcanizates shown in Figure 3, the glass transition of NR elastomer occurred with the T_g_ in the range of −74 °C to −69 °C. The values of T_g_ obtained using DMA were lower than those determined by DSC (Table 4) due to the different conditions of measurements. During DSC measurement, a small piece of the uncured rubber compound was only exposed to temperature, whereas in DMA, analysis vulcanized samples were treated with both temperature and oscillating strain. Thus, the T_g_ in DMA measurements was determined in dynamic conditions.

The T_g_ of NR determined for the benchmark without filler was of approximately −69 °C, whereas the T_g_ for the silica-filled vulcanizate was −72 °C. Taking into account the measurement error, ILs did not significantly affect the T_g_ of the silica-filled NR elastomer determined in the dynamic conditions. Thus, the addition of ILs, regardless of their structure, should not influence the operating temperature of NR composites in dynamic conditions, i.e., under oscillating deformations.

Mechanical loss factor (tan δ) is a measure of the material’s ability to dampen vibrations. As expected, the highest value of tan δ at T_g_ (Table 7) of approximately 2.1 was exhibited by the unfilled vulcanizate. It resulted from the highest flexibility of this vulcanizate and the highest mobility of the elastomer chains in the unfilled elastomer network. The addition of silica reduced twice the height of the tan δ peak (Figure 3) as compared to the unfilled benchmark, which was due to the significantly decreased elastomer chains mobility by the filler’s network [69]. Active fillers, such as silica, form their own network in the crosslinked elastomer matrix, increasing the stiffness and hence reducing the flexibility of the NR vulcanizates and therefore the values of tan δ at T_g_. The same effect of the silica on the damping properties of vulcanizates was reported by Yoon et al. [70]. Most of the ILs reduced the tan δ in T_g_ (Figure 3a,b), which resulted from the higher crosslink density of vulcanizates compared to NR composite without ILs. It is commonly known that the mobility of the elastomer chains in the crosslinked elastomer network decreases when the number of crosslinks increases. The structure of ILs did not have a remarkable influence on the changes in the tan δ at T_g_.

Regarding the tan δ at 25 °C and 50 °C, i.e., in the rubbery elastic region, the addition of the nanosized silica significantly increased tan δ (Table 7) compared to the unfilled NR, whereas ILs did not considerably affect this parameter as compared to the silica-filled vulcanizate. The obtained values of the tan δ showed that NR vulcanizates filled with the nanosized silica and containing ILs were characterized by a good capacity to dampen vibrations and had stable dynamic properties in the rubbery elastic region.

### 3.4. Thermo-Oxidative Aging Resistance of NR Composites Filled with Nanosized Silica

**Figure 4:**
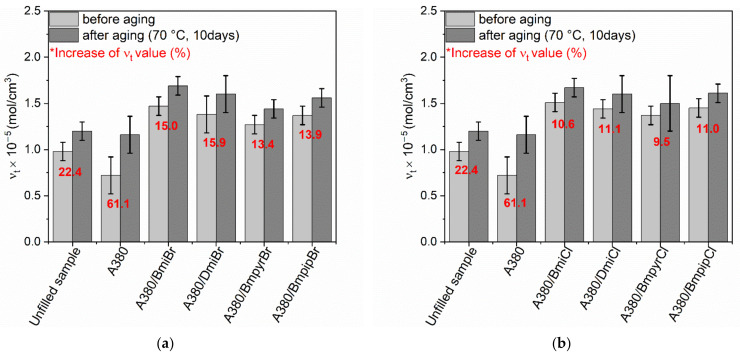
Influence of thermo-oxidative aging on the crosslink density of the silica-filled NR vulcanizates containing ILs with: (**a**) bromide anion; (**b**) chloride anion.

**Figure 5:**
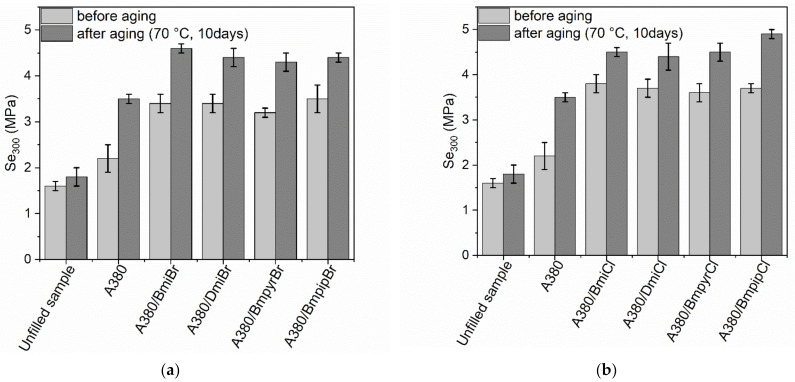
Influence of thermo-oxidative aging on the stress at 300% relative elongation (Se_300_) of the silica-filled NR vulcanizates containing ILs with: (**a**) bromide anion; (**b**) chloride anion.

**Figure 6:**
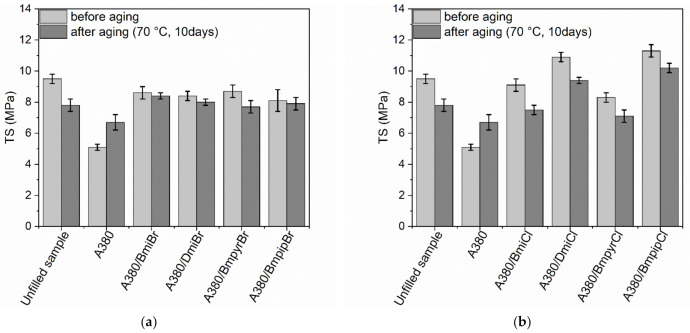
Influence of thermo-oxidative aging on the tensile strength (TS) of the silica-filled NR vulcanizates containing ILs with: (**a**) bromide anion; (**b**) chloride anion.

**Figure 7:**
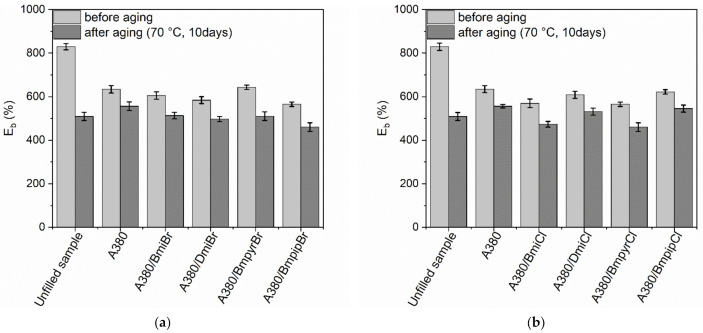
Influence of thermo-oxidative aging on the elongation at break (E_b_) of the silica-filled NR vulcanizates containing ILs with: (**a**) bromide anion; (**b**) chloride anion.

**Figure 8:**
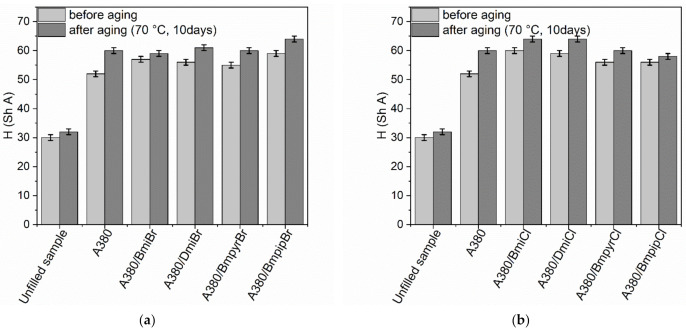
Influence of thermo-oxidative aging on the hardness of the silica-filled NR vulcanizates containing ILs with: (**a**) bromide anion; (**b**) chloride anion.

It is known that NR composites are characterized with poor aging resistance as compared to most synthetic rubbers [11]. Hence, it is desirable from a technological viewpoint that the ILs will not further deteriorate the resistance of NR vulcanizates to prolonged thermo-oxidation. Therefore, the impact of ILs on the resistance of NR vulcanizates to thermo-oxidative aging was determined in the next step of this work. NR vulcanizates were stored at 70 °C for 240 h, and then their crosslink density, hardness and tensile properties were tested and compared with the same properties of non-aged vulcanizates. The influence of prolonged thermo-oxidation on the properties of NR composites is shown in Figure 4, Figure 5, Figure 6, Figure 7 and Figure 8.

As expected, prolonged exposure to elevated temperature during thermo-oxidative aging resulted in further crosslinking of NR composites, especially those filled with nanosized silica. Consequently, the *ν_t_* of NR vulcanizates after thermo-oxidative aging was higher compared to that of non-aged samples. A similar influence of thermo-oxidative aging on the *ν_t_* of elastomer composites was reported by Choe et al. [60], who explained that the change in the crosslink densities of vulcanizates resulting from the prolonged exposure to elevated temperature was due to the dissociation of the existing sulfur crosslink and the formation of new crosslinks by free sulfur, pendent sulfide groups and products of curing agents’ reactions [71]. In the case of the unfilled benchmark, a significantly smaller increase in the *ν_t_* was achieved compared to the silica-filled vulcanizates. This was probably due to the higher vulcanization efficiency of the unfilled NR compound compared to the silica-filled composite and the consequently higher consumption of the curing system. Owing to the adsorption of the curing system components on the silica surface, their consumption during vulcanization and, consequently, the efficiency of the vulcanization was lower than that of the unfilled NR compound. However, prolonged exposure to elevated temperatures induced the desorption of the unconsumed curatives from the silica surface. This facilitated the further crosslinking of the silica-filled NR composites during thermo-oxidative aging, since desorbed curatives can participate in the crosslinking reactions to make new crosslinks. This was confirmed by Choi et al. [72] for the silica-filled SBR composites. The addition of ILs significantly reduced the percentage increase in *ν_t_* compared to the vulcanizate without ILs (Figure 4). The structure of ILs affected the changes in the crosslink density of the vulcanizates resulting from the prolonged thermo-oxidation. Vulcanizates containing ILs with chloride anion were characterized by a considerably lower increase in the *ν_t_* due to thermo-oxidative aging compared to those containing bromide ILs. Regarding the influence of cation, in the case of bromides, slightly higher changes in *ν_t_* were observed for imidazolium ILs. In the case of chlorides, the effect of cation on *ν_t_* increase was smaller compared to bromides. No unequivocal relationship was observed between the type of ILs cation and the increase in *ν_t_* due to thermos-oxidative aging.

The influence of thermo-oxidative aging on the stress at 300% relative elongation (Se_300_) of the vulcanizates was shown in Figure 5. It is known that Se_300_ strongly depends on the *ν_t_* of the vulcanizates. Therefore, the influence of thermo-oxidative aging on the Se_300_ of NR vulcanizates corresponded to changes in the *ν_t_*. Thus, the unfilled vulcanizate exhibited slightly higher Se_300_ compared to the non-aged sample. On the other hand, the silica-filled vulcanizates showed significantly higher Se_300_ after thermo-oxidative aging compared to non-aged samples, which resulted from the significant increase in their crosslink density. Moreover, according to Debnath et al. [73], during prolonged exposure to high temperatures, the polysulfide linkages in the elastomer network are broken down and then monosulfide linkages form, which increase the Se_300_ modulus of elastomer material.

An increase in the *ν_t_* due to thermo-oxidative aging had a significant influence on the TS (Figure 6) and E_b_ (Figure 7) of the silica-filled NR vulcanizates.

Prolonged exposure to elevated temperatures deteriorated the TS of both the unfilled benchmark and the silica-filled vulcanizates containing ILs. Further crosslinking, induced by the thermo-oxidative aging process, resulted in these vulcanizates being over-crosslinked and thus brittle and less resistant to mechanical stress [74]. The structure—and, more precisely, the type of anion—of ILs affected the changes in TS due to the thermo-oxidative aging. Vulcanizates with ILs bearing bromide anion, especially those containing imidazolium cation, showed significantly smaller changes in TS (Figure 6a) as compared to vulcanizates containing ILs with chloride anion (Figure 6b). On the other hand, the silica-filled vulcanizate without ILs exhibited considerably higher TS after prolonged thermo-oxidation compared to non-aged vulcanizate. It should be noticed that this vulcanizate exhibited a significantly lower *ν_t_* before aging compared to other vulcanizates. Thus, further crosslinking of this vulcanizate during thermo-oxidative aging did not result in over-crosslinking but improved the TS of the material. It is commonly known that the TS of the vulcanizates, which are not over-crosslinked during vulcanization, increases when the crosslink density increases [66].

Most of the silica-filled vulcanizates exhibited approximately 100% lower E_b_ (Figure 7) as compared to non-aged vulcanizates. This was due to the increase in their crosslink density resulting from thermo-oxidative aging. On the other hand, the thermo-oxidative aging process had a great influence on the E_b_ of the unfilled benchmark, which exhibited approximately 300% lower E_b_ compared to the non-aged sample. The addition of ILs and their structure did not significantly affect the changes in E_b_ resulting from thermo-oxidative aging.

Prolonged exposure to elevated temperatures increased the hardness (H) of the unfilled NR vulcanizate by approximately 3 Shore A (Figure 8). On the other hand, the hardness of the silica-filled vulcanizate without ILs was of approximately 9 Shore A higher compared to the non-aged sample. This resulted from the significant increase in the crosslink density of this vulcanizates due to thermo-oxidative aging. The prolonged thermo-oxidation hardness of the ILs-containing vulcanizates was of approximately 2–5 Shore A higher compared to the non-aged samples.

The aging factor (A_f_) was considered as a measure of material’s resistance to thermo-oxidative aging. A_f_ was calculated using the changes in the TS and E_b_ of vulcanizates, which resulted from the aging process. Equation (3), presented earlier, was used for A_f_ calculations. The value of A_f_ close to 1 means that the aging process did not considerably affect the tensile properties of the vulcanizates and thus the material is resistant to thermo-oxidative aging. The results are given in Table 8.

The unfilled benchmark exhibited an A_f_ of approximately 0.5, which resulted from the significant reduction in the TS and E_b_ of this vulcanizate due to aging. Thus, it was susceptible to prolonged thermo-oxidation. The application of nanosized silica resulted in an approximately 2-fold increase in A_f_ as compared to the unfilled benchmark, which was due to the significant improvement in the TS of the silica-filled NR composite resulting from the aging. On the other hand, the addition of ILs reduced the A_f_ compared to the vulcanizate without ILs. However, the values of A_f_ for vulcanizates with ILs ranged from 0.7 to 0.9; so they were still much higher than the A_f_ of the unfilled vulcanizate. Most importantly, NR vulcanizates containing nanosized silica and ILs exhibited significantly better resistance to thermo-oxidative aging compared to the unfilled NR.

### 3.5. Effect of Ionic Liquids on the Thermal Stability of NR Composites Filled with Nanosized Silica

**Figure 9:**
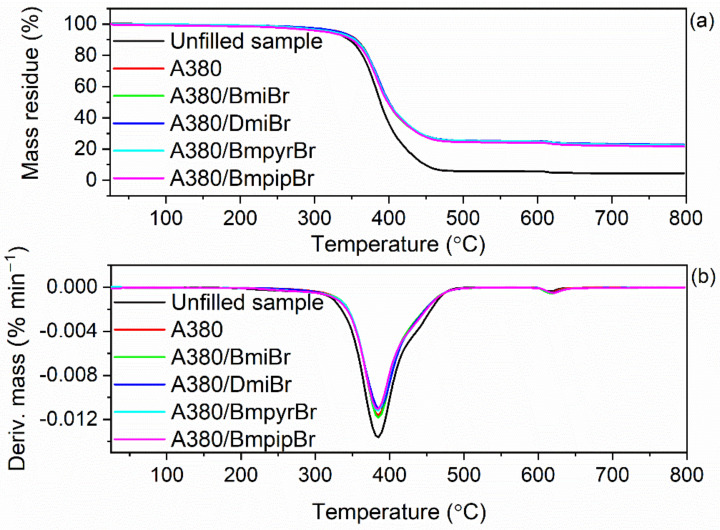
Thermogravimetric (TG) and derivative thermogravimetric (DTG) curves of the silica-filled NR vulcanizates containing ILs with bromide anion: (**a**) TG curves; (**b**) DTG curves.

**Figure 10:**
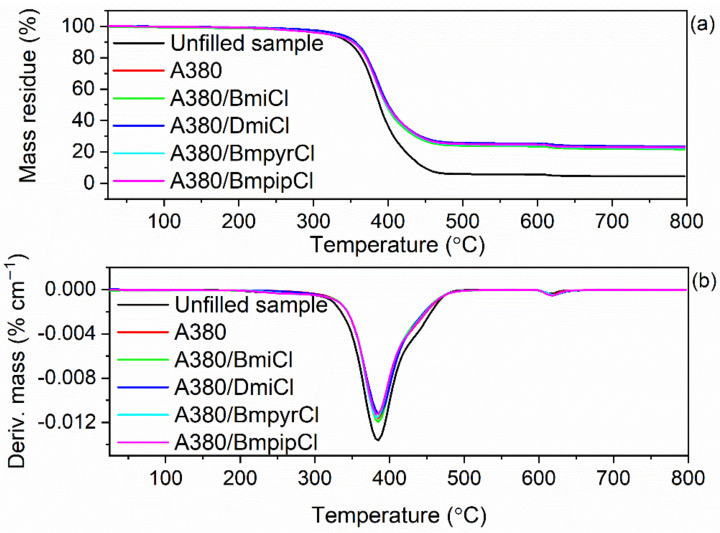
TG and DTG curves of the silica-filled NR vulcanizates containing ILs with chloride anion: (**a**) TG curves; (**b**) DTG curves.

Both the elastomer matrix and the additives, especially organic compounds, can decompose when heated to high temperatures, which affects the thermal stability of elastomer composites. It is known that pure ILs exhibit different thermal stability, which is mainly dependent on the structure of their cation [41,56,64,75]. Therefore, it was reasonable to explore the impact of ILs and their structure on the thermal stability of NR vulcanizates. The results of thermogravimetry (TG) are summarized in Table 9 and plotted in Figure 9 and Figure 10.

TG analysis was performed in two stages. The first stage of measurements was performed in a temperature range of 25–600 °C in an inert gas-argon. Thus, a pyrolysis of elastomer and other organic components of NR composites, i.e., stearic acid, vulcanization accelerator and ILs, proceeded in this stage. The mass loss at 25–600 °C for the unfilled vulcanizate was of approximately 94.6%, significantly higher than for the silica-filled vulcanizates (mass loss of approximately 75.2%). This resulted from the higher rubber content in the same amount of rubber compound compared to vulcanizates filled with 30 phr of the nanosized silica. Vulcanizates containing ILs were characterized by a mass loss similar to the silica-filled vulcanizate without the ionic liquid. The next stage of measurement was performed in a temperature range of 600–800 °C in an air atmosphere; so, combustion of the residue after thermal decomposition of the vulcanizates proceeded in this stage with a mass loss of approximately 2%. The residue after thermal decomposition of the material at 800 °C was dependent on the composition of the NR composite. Regarding the unfilled benchmark, the residue at 800 °C was 4.2%, which mainly resulted from the use of zinc oxide as a vulcanization activator. Considering vulcanizates filled with nanosized silica, the residue at 800 °C was due to the presence of both zinc oxide and nanosized silica. Regardless of the composition of the silica-filled vulcanizates, the residue at 800 °C was approximately 22%, because the amount of activator and nanosized silica used was the same in all composites.

The application of nanosized silica and ILs significantly affected the onset decomposition temperature (T_5%_) of the vulcanizates, whereas no meaningful effect on the temperature of the maximum mass loss rate (T_DTG_) was observed. The unfilled benchmark began to thermally decompose at a temperature of approximately 317 °C (Table 8). The addition of nanosized silica improved the thermal stability of NR composites compared to the unfilled one. The silica-filled vulcanizate exhibited a T_5%_ of 333 °C, approximately 16 °C higher than the unfilled benchmark, which resulted from the high thermal stability of silica itself [76]. Moreover, the network of silica nanoparticles dispersed in the elastomer matrix formed a barrier to the diffusion of gases and gaseous products of thermal decomposition throughout the elastomer composite, increasing the T_5%_ [77]. The influence of ILs on the thermal stability of NR vulcanizates depended on their structure, mainly on the type of cation. This was due to the strong dependence of the thermal stability of ILs on their structure and, in particular, the type of cation. According to Xue et al. [75] imidazolium salts showed higher thermal stability than ILs with other cations. We have also confirmed that the presence of the unsaturated heterocyclic imidazolium ring in the cation facilitated the thermal stability compared to ILs having a saturated heterocyclic ring, i.e., a pyrrolidinium or piperidinium ring [56,64]. Thus, ILs with pyrrolidinium and piperidinium cation reduced the T_5%_ by 12–20 °C as compared to the vulcanizate without ILs. However, vulcanizates with pyrrolidinium and piperidinium ILs exhibited T_5%_ in the range of 313–321 °C, still comparable to the unfilled benchmark. Nanosized silica and ILs had no considerable influence on the T_DTG_—the temperature at which the thermal decomposition of the material proceeded with the highest rate. Most importantly, ILs did not deteriorate the thermal stability as compared to the unfilled NR composites. Therefore, nanosized silica and ILs should not limit or diminish the potential technological uses of NR composites as compared to unfilled ones.

## 4. Conclusions

In this work, the influence of the structure, i.e., the type of cation (imidazolium, pyrrolidinium and piperidinium), and the type of anion (bromide and chloride) on the curing characteristics and performance of natural rubber (NR) composites filled with nanosized silica was investigated. Most importantly, the ILs tested were proved to have a beneficial influence on the vulcanization parameters of the NR composites filled with silica nanoparticles and consequently on the performance of vulcanizates as compared to that without ILs. This is very important for the potential use of such biocomposites.

Nanosized silica increased the viscosity of the uncrosslinked NR compound as compared to the unfilled benchmark. ILs reduced the viscosity, which facilitated the processing of rubber compounds. Owing to the adsorption of the curing system, nanosized silica significantly increased the optimal vulcanization time of NR compounds and reduced the crosslink density of the vulcanizates. Regardless of their structure, ILs shortened the time of vulcanization and reduced the onset vulcanization temperature to values comparable to the unfilled benchmark. Moreover, ILs enhanced the efficiency of vulcanization and consequently significantly increased the crosslink density of vulcanizates compared to the unfilled benchmark and the silica-filled vulcanizate without ILs. The ILs were proved to support the vulcanization of the NR composites filled with silica nanoparticles.

The structure of ILs, especially the type of cation, affected their activity in the vulcanization and thus the crosslink density of the vulcanizates. NR composites containing imidazolium ILs, especially with butyl substituent, were characterized by a higher crosslink density as compared to vulcanizates with pyrrolidinium and piperidinium ILs. The anion of ILs had no significant influence on their activity in vulcanization, although chlorides seemed to be slightly more active compared to ILs with bromide anion.

Due to the beneficial influence on the crosslink density, the addition of ILs caused a significant improvement in the tensile strength of NR vulcanizates filled with nanosized silica. On the other hand, ILs had no considerable effect on the damping properties of NR vulcanizates.

Applying nanosized silica and ILs enhanced the resistance of NR vulcanizates to thermo-oxidative aging and improved the thermal stability compared to the unfilled composite. The thermal stability of the silica-filled vulcanizates was dependent on the structure of ILs, especially the type of the cation. The most thermally stable were NR vulcanizates containing imidazolium ILs due to the highest thermal stability of imidazolium salts as compared to those with pyrrolidinium and piperidinium cation.

Most importantly, the performed studies proved that the application of nanosized silica together with the ILs of various structures allowed for the obtention of NR biocomposites characterized by improved curing characteristics and crosslink density, good processing properties, satisfied mechanical performance, high thermal stability and a resistance to prolonged thermo-oxidation.

## Figures and Tables

**Table 1:** Ionic liquids (ILs) used in the natural rubber (NR) compounds.

Name	Abbreviation	CAS Number	Purity (%)	Water Content (wt. %)
Imidazolium ionic liquids				
1-butyl-3-methylimidazolium bromide	BmiBr	85100-77-2	≥99.0%	<0.5%
1-butyl-1-methylimidazolium chloride	BmiCl	79917-90-1	≥99.0%	≤0.2%
1-decyl-3-methylimidazolium bromide	DmiBr	188589-32-4	>98%	<1%
1-decyl-3-methylimidazolium chloride	DmiCl	171058-18-7	96.0%	≤2.0%
Pyrrolidinium ionic liquids				
1-butyl-1-methylpyrrolidinium bromide	BmpyrBr	93457-69-3	≥99.0%	≤0.5%
1-butyl-1-methylpyrrolidinium chloride	BmpyrCl	479500-35-1	≥99.0%	≤0.5%
Piperidinium ionic liquids				
1-butyl-1-methylpiperidinium bromide	BmpipBr	94280-72-5	99%	0.5%
1-butyl-1-methylpiperidinium chloride	BmpipCl	845790-13-8	99%	<1%

**Table 2:** General recipes of natural rubber (NR) compounds, mass of ingredients given in parts per hundred of rubber (phr); (MBT, 2-mercaptobenzothiazole; St.A., stearic acid; IL, ionic liquid).

Ingredient, phr	Unfilled Sample (R1)	NR/A380 (R2)	NR/A380/IL (R3–R10)
NR	100	100	100
Sulfur	2	2	2
ZnO	5	5	5
MBT	2	2	2
St.A.	1	1	1
Silica A380	₋	30	30
IL *	₋	₋	3

* In rubber compounds, R3–R10 ionic liquids given in Table 1 were used.

**Table 3:** Cure characteristics at 160 °C of NR compounds filled with nanosized silica (S_min_, minimum torque; S_max_, maximum torque; ∆S, torque increase; t_02_, scorch time; t_90_, optimal vulcanization time; standard deviations: S_min_ ± 0.4 dNm, S_max_ ± 1.1 dNm, ΔS ± 1.1 dNm, t_02_ ± 0.2 min, t_90_ ± 0.3 min).

Compounds	S_min_ (dNm)	S_max_ (dNm)	∆S (dNm)	t_02_ (min)	t_90_ (min)
Unfilled sample	0.3	5.0	4.7	1	2
NR compound without ionic liquid					
A380	6.0	11.4	5.4	2	12
NR compounds with imidazolium ionic liquids					
A380/BmiBr	6.1	13.5	7.4	1	2
A380/DmiBr	5.8	15.0	9.2	1	3
A380/BmiCl	5.8	14.5	8.7	1	2
A380/DmiCl	5.4	16.8	11.8	1	3
NR compounds with pyrrolidinium ionic liquids					
A380/BmpyrBr	6.7	15.2	8.5	1	3
A380/BmpyrCl	5.6	12.4	6.8	1	1
NR compounds with piperidinium ionic liquids					
A380/BmpipBr	6.9	13.8	6.9	1	2
A380/BmpipCl	6.4	13.6	7.2	1	2

**Table 4:** Differential scanning calorimetry (DSC) of NR compounds filled with nanosized silica (T_g_, glass transition temperature; ∆C_p_, change in the heat capacity; ∆H_cross_, enthalpy of crosslinking; T_g_ ± 1 °C, ∆C_p_ ± 0.1 J/g × K, temperature ± 6.0 °C; ΔH ± 1.3 J/g).

Compounds	T_g_ (°C)	∆C_p_ (J/g × K)	Temperature of Crosslinking (°C)	∆H_cross_ (J/g)
Unf illed sample	−64.2	0.44	146–208	9.0
NR compound without ionic liquid				
A380	−63.6	0.31	171–211	10.1
NR compounds with imidazolium ionic liquids				
A380/BmiBr	−63.6	0.37	154–190	7.0
A380/DmiBr	−64.1	0.37	157–197	12.7
A380/BmiCl	−63.9	0.32	151–190	7.9
A380/DmiCl	−64.3	0.31	156–194	10.5
NR compounds with pyrrolidinium ionic liquids				
A380/BmpyrBr	−63.5	0.30	154–195	11.0
A380/BmpyrCl	−63.0	0.30	150–189	9.1
NR compounds with piperidinium ionic liquids				
A380/BmpipBr	−63.2	0.30	153–194	11.4
A380/BmpipCl	−63.4	0.29	150–191	9.8

**Table 5:** Equilibrium swelling in toluene (Q_t_) and crosslink density (*ν_t_*) of the silica-filled NR vulcanizates containing ILs (SD: Q_t_ ± 0.4; *ν_t_* ± 0.3 mol/cm^3^ × 10^−5^).

NR Vulcanizates	Q_t_ (−)	*ν_t_* × 10^−5^ (mol/cm^3^)
Unfilled sample	4.47	0.98
NR vulcanizate without ionic liquid		
A380	5.41	0.72
NR vulcanizates with imidazolium ionic liquids		
A380/BmiBr	3.58	1.47
A380/DmiBr	3.71	1.38
A380/BmiCl	3.53	1.51
A380/DmiCl	3.62	1.44
NR vulcanizates with pyrrolidinium ionic liquids		
A380/BmpyrBr	3.87	1.37
A380/BmpyrCl	3.72	1.37
NR vulcanizates with piperidinium ionic liquids		
A380/BmpipBr	3.72	1.37
A380/BmpipCl	3.70	1.40

**Table 6:** Mechanical properties and hardness of the silica-filled NR vulcanizates containing ILs (SE_100_, stress at 100% relative elongation; SE_300_, stress at 300% relative elongation; TS, tensile strength; EB, elongation at break; H, hardness).

NR Vulcanizates	Se_100_ (MPa)	Se_300_ (MPa)	TS ()	E_b_ (%)	H (Shore A)
Unfilled sample	1.1 ± 0.1	1.6 ± 0.1	9.5 ± 0.4	829 ± 14	30 ± 1
	NR vulcanizate without ionic liquid				
A380	1.6 ± 0.1	2.2 ± 0.1	5.1 ± 0.3	634 ± 18	52 ± 1
	NR vulcanizates with imidazolium ionic liquids				
A380/BmiBr	1.8 ± 0.1	3.4 ± 0.1	8.6 ± 0.2	605 ± 20	57 ± 1
A380/DmiBr	1.7 ± 0.1	3.4 ± 0.1	8.4 ± 0.3	584 ± 9	56 ± 1
A380/BmiCl	1.8 ± 0.1	3.8 ± 0.1	9.1 ± 0.8	569 ± 16	60 ± 1
A380/DmiCl	1.8 ± 0.1	3.7 ± 0.1	10.9 ± 0.6	608 ± 11	59 ± 1
	NR vulcanizates with pyrrolidinium ionic liquids				
A380/BmpyrBr	1.7 ± 0.1	3.2 ± 0.1	8.7 ± 0.5	643 ± 6	55 ± 1
A380/BmpyrCl	1.8 ± 0.1	3.6 ± 0.1	8.3 ± 0.8	565 ± 11	56 ± 1
	NR vulcanizates with piperidinium ionic liquids				
A380/BmpipBr	1.8 ± 0.1	3.5 ± 0.1	8.1 ± 0.9	578 ± 19	59 ± 1
A380/BmpipCl	1.8 ± 0.1	3.7 ± 0.1	11.3 ± 0.7	622 ± 14	56 ± 1

**Table 7:** Glass transition temperature (T_g_) determined by the dynamic mechanical analysis (DMA) and mechanical loss factor (tan δ) of the silica-filled NR vulcanizates containing ILs.

NR Vulcanizates	T_g_ (°C)	Tan δ_Tg_ (−)	Tan δ_25 °C_ (−)	Tan δ_50 °C_ (−)
Unfilled sample	−69 ± 1	2.10 ± 0.1	0.05 ± 0.02	0.04 ± 0.02
NR vulcanizate without ionic liquid				
A380	−72 ± 1	1.10 ± 0.1	0.13 ± 0.02	0.13 ± 0.02
NR vulcanizates with imidazolium ionic liquids				
A380/BmiBr	−74 ± 1	0.81 ± 0.1	0.11 ± 0.01	0.11 ± 0.01
A380/DmiBr	−72 ± 1	0.90 ± 0.1	0.11 ± 0.01	0.10 ± 0.01
A380/BmiCl	−73 ± 1	0.79 ± 0.1	0.10 ± 0.02	0.11 ± 0.02
A380/DmiCl	−70 ± 1	0.90 ± 0.1	0.10 ± 0.02	0.10 ± 0.02
NR vulcanizates with pyrrolidinium ionic liquids				
A380/BmpyrBr	−73 ± 1	0.90 ± 0.1	0.10 ± 0.01	0.10 ± 0.01
A380/BmpyrCl	−74 ± 1	0.89 ± 0.1	0.11 ± 0.02	0.13 ± 0.02
NR vulcanizates with piperidinium ionic liquids				
A380/BmpipBr	−74 ± 1	0.91 ± 0.1	0.10± 0.01	0.10 ± 0.01
A380/BmpipCl	−71 ± 1	0.91 ± 0.1	0.09 ± 0.01	0.10 ± 0.01

**Table 8:** Thermo-oxidative aging factor (A_f_) of the silica-filled NR vulcanizates containing ILs.

SBR Vulcanizates	A_f_ (−)
Unfilled sample	0.5 ± 0.1
NR vulcanizate without ionic liquid	
A380	1.2 ± 0.1
NR vulcanizates with imidazolium ionic liquids	
A380/BmiBr	0.8 ± 0.1
A380/DmiBr	0.8 ± 0.1
A380/BmiCl	0.7 ± 0.1
A380/DmiCl	0.8 ± 0.1
NR vulcanizates with pyrrolidinium ionic liquids	
A380/BmpyrBr	0.7 ± 0.1
A380/BmpyrCl	0.7 ± 0.1
NR vulcanizates with piperidinium ionic liquids	
A380/BmpipBr	0.9 ± 0.1
A380/BmpipCl	0.8 ± 0.1

**Table 9:** Onset temperature of thermal decomposition (T_5%_), DTG peak temperature (T_DTG_) and total mass loss (∆m) during decomposition of the silica-filled NR composites containing ILs (SD: T_5%_ ± 1.2 °C; T_DTG_ ± 1.3 °C; ∆m ± 1.3%).

SBR Vulcanizates	T_5%_ (°C)	T_DTG_ (°C)	∆m_25–600 °C_ (%)	∆m_600–800 °C_ (%)	Residue at 800 °C (%)
Unfilled sample	317	401	94.0	1.8	4.2
NR vulcanizate without ionic liquid					
A380	333	401	75.2	1.9	22.9
NR vulcanizates with imidazolium ionic liquids					
A380/BmiBr	329	402	75.3	2.4	22.3
A380/DmiBr	333	403	74.9	2.2	22.9
A380/BmiCl	327	402	75.9	2.0	22.1
A380/DmiCl	333	403	75.1	2.0	22.9
NR vulcanizates with pyrrolidinium ionic liquids					
A380/BmpyrBr	321	401	75.1	2.3	22.6
A380/BmpyrCl	321	400	75.7	2.1	22.2
NR/A380 vulcanizates with piperidinium ionic liquids					
A380/BmpipBr	315	401	75.3	2.3	22.4
A380/BmpipCl	317	400	75.3	2.0	22.7

## Data Availability

The data presented in this study are available on request from the corresponding author.
